# Effect of Joint Mobilization in Individuals with Chronic Ankle Instability: A Systematic Review and Meta-Analysis

**DOI:** 10.3390/jfmk7030066

**Published:** 2022-09-06

**Authors:** Hyunjoong Kim, Seoyoung Moon

**Affiliations:** 1 Neuromusculoskeletal Science Laboratory, Gwangju 62287, Korea; 2 Department of Research, Good Morning Nursing Hospital, Gwangju 61102, Korea

**Keywords:** ankle injury, manual therapy, physical therapy, postural balance, joint range of motion

## Abstract

Sensorimotor and range of motion deficits due to chronic ankle instability (CAI) are abnormalities of the movement system that make postural control difficult. This review aimed to quantify the effect of joint mobilization on the range of motion, dynamic balance, and function in individuals with CAI. Randomized controlled trials in which joint mobilization was performed in individuals with CAI were searched for in five international databases (CENTRAL, CINAHL, Embase, MEDLINE, PEDro). Qualitative and quantitative analyses were performed using the risk of bias tool and RevMan 5.4 provided by the Cochrane Library. Nine studies with 364 individuals with CAI were included in this study. This meta-analysis reported that joint mobilization showed significant improvement in the dorsiflexion range of motion (standardized mean difference [SMD] = 1.02, 95% confidence interval [CI]: 0.41 to 1.63) and dynamic balance (SMD = 0.49, 95% CI: 0.06 to 0.78) in individuals with CAI. However, there was no significant improvement in function (patient-oriented outcomes) (SMD = 0.76, 95% CI: −0.00 to 1.52). For individuals with CAI, joint mobilization has limited function but has positive benefits for the dorsiflexion range of motion and dynamic balance.

## 1. Introduction

Among musculoskeletal injuries, ankle sprains are common, with a high recurrence rate and persistent symptoms [1,2]. This causes chronic ankle instability (CAI) accompanied by pain and swelling along with muscle weakening around the ankle joint [3,4].

Sensorimotor and range of motion (ROM) deficits due to CAI lead to altered movement strategies [5]. Therefore, it is reported that there are movement system abnormalities in various physical activities, and in a systematic review by Rosen et al. [6], it was also reported that individuals with CAI had a deficiency in static and dynamic postural control. It affects the nervous system that controls movement and is explained by the loss of joint position sensation due to damage to the receptor and peroneal nerve caused by ankle ligament injury [7,8,9]. In a study of muscle activity patterns, the onset of muscle activity in the tibialis anterior and peroneus longus was longer in patients with functional ankle instability compared to healthy adults [10]. These results indicate that ankle instability is defective in the peripheral reflex stability of the ankle [11].

According to the clinical practice guidelines of Martin et al. [5], therapeutic exercise, activity, and manual therapy are recommended as strong evidence. Among the manual therapies frequently used in physical therapy clinics, joint mobilization (JM) has been reported to provide strong evidence for the improvement of dynamic postural control; furthermore, significant improvements in pain, patient-oriented outcomes, and ROM were reported in all sub-techniques (manipulations, non-weight-bearing, and weight-bearing mobilization) [12,13].

Among the various treatments for CAI, we focused on JM as the core treatment. Therefore, in this review, randomized controlled trials (RCTs) were synthesized to investigate the effect of JM on individuals with CAI. A systematic review and meta-analysis were performed with qualitative and quantitative analysis on the effect of JM through the synthesized data.

## 2. Materials and Methods

### 2.1. Study Design

This review is a systematic review and meta-analysis of randomized controlled trials (RCTs) in which JM was performed on CAI individuals. The systematic review was prepared according to the Preferred Reporting Items for Systematic Reviews and Meta-Analysis (PRISMA) guidelines. Before the review, the protocol was registered in the Prospective Register of Systematic Reviews (PROSPERO) (No.: CRD42020220149).

### 2.2. Search Strategy and Selection of Studies

#### 2.2.1. Inclusion Criteria

1. Participants

Participants were individuals with CAI enrolled in RCTs.

2. Intervention

Studies in which JM was applied in manual therapy were included.

3. Comparisons

To determine the effectiveness of JM alone, interventions without JM were included if no interventions were provided or if there were a combined intervention.

4. Outcomes

For quantitative comparison, we included outcome measures with three or more common variables.

5. Types of studies

In this review, we included RCTs extracted from the database.

#### 2.2.2. Exclusion Criteria

Studies that could not compare the effects of JM alone, studies prior to 2013, and studies other than English were excluded.

#### 2.2.3. Literature Search Strategy

Studies were collected in July 2022 after registration in PROSPERO for this review. Each was searched by researchers with meta-analysis experience. The search keywords consisted of the following terms: joint mobilization, chronic ankle instability, and a randomized controlled trial.

The international electronic databases used for the search were the Cochrane Central Register of Controlled Trials (CENTRAL), Cumulative Index to Nursing and Allied Health Literature (CINAHL), Excerpta Medica Database (Embase), Medical Literature Analysis and Retrieval System Online (MEDLINE), and Physiotherapy Evidence Database (PEDro).

#### 2.2.4. Study Selection and Data Extraction

For the studies extracted from the database, duplicate data were removed using a reference management tool (EndNote 20, Thomson Reuters, New York, NY, USA). In accordance with the PRISMA guidelines, the title and abstract were reviewed to continue the full text review. Afterwards, the researchers explained the extracted and non-extracted studies, respectively, and went through the process of classifying the selected studies and extracting features.

#### 2.2.5. Quality Assessment

A quality assessment was conducted using the Cochrane Risk of Bias (RoB) tool to evaluate the included RCTs. The RoB consists of 7 items (random sequence generation, allocation concealment, blinding of participants and personnel, blinding of outcome assessment, incomplete outcome data, selective reporting, other bias) and was evaluated as low (+), uncertain (?), or high (−) by the researchers. In cases of disagreement, a consensus was reached through an agreement process.

### 2.3. Strategy for Data Synthesis

RevMan 5.4 (The Cochrane Collaboration, Oxford, UK) was used to synthesize the included studies. A meta-analysis was performed using outcome measures for which the quantitative values were provided in the included studies. When there were three or more identical outcome measures, it was selected as an outcome measure and extracted through standard mean difference (SMD). For the effect model, a random effect model considering heterogeneity was adopted [14].

For the heterogeneity of RCTs, the chi-square test and I^2^ test provided in RevMan 5.4 were used. If the value of I^2^ is more than 75%, the heterogeneity is high, and if it is less than 40%, the heterogeneity is considered low [15]. The publication bias of the synthesized studies is shown through a funnel plot [16].

## 3. Results

### 3.1. Literature Search and Characteristics of the Included Randomized Clinical Trials

**Figure 1:**
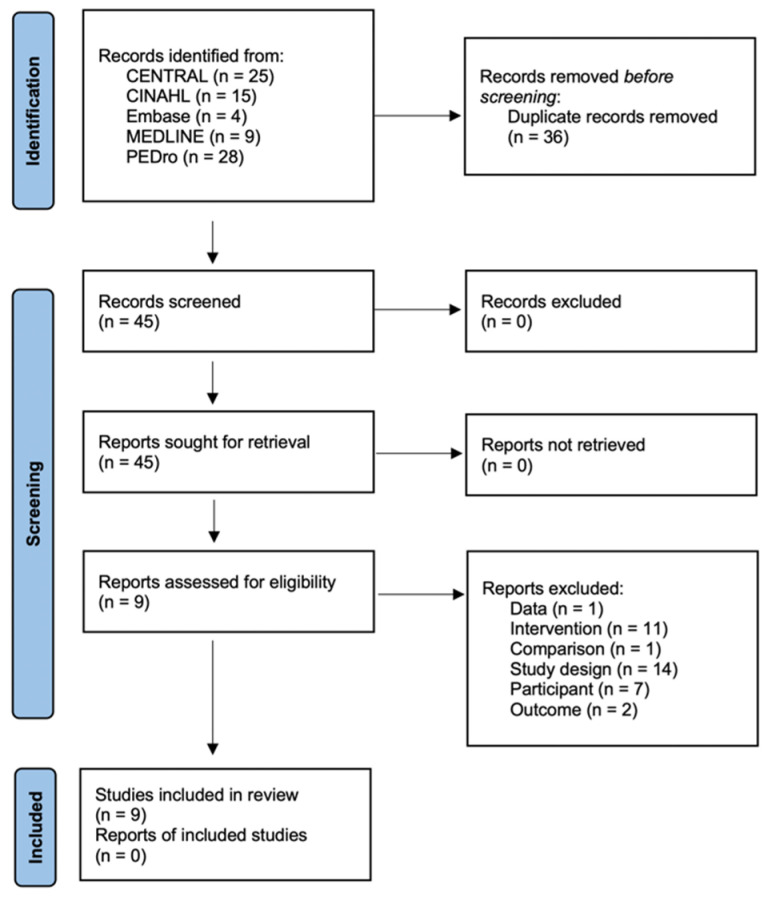
PRISMA flow diagram.

A total of 81 papers were searched through five electronic databases. For duplicate papers, 36 were excluded through the reference management tool. As shown in Figure 1, 36 studies were excluded because of the inclusion criteria. Therefore, in this review, nine papers were selected [17,18,19,20,21,22,23,24,25], and a systematic review and meta-analysis were conducted (Figure 1).

### 3.2. Methodological Quality Assessment

**Figure 2:**
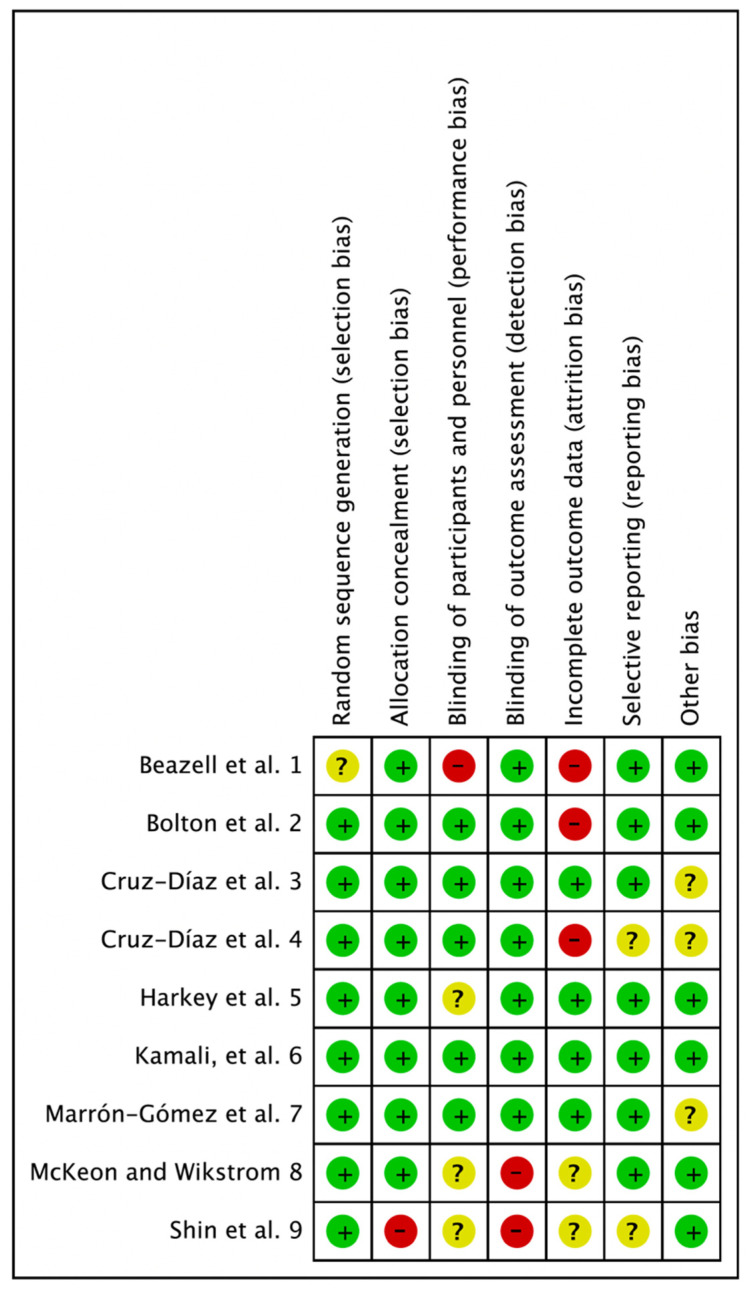
Risk of bias summary: review of authors’ judgements about each risk of bias item for each included study. 1—[18], 2—[23], 3—[19], 4—[17], 5—[25], 6—[24], 7—[22], 8—[21], 9—[20].

A pilot test was conducted to arrive at a consensus result. The results obtained when the coincidence rate was 100% are as follows: random sequence generation (low: eight; uncertain: one), allocation concealment (low: eight; high: one), blinding of participants and personnel (low: five; uncertain: three, high: one), blinding of outcome assessment (low: seven; high: two), incomplete outcome data (low: four; uncertain: two; high: three), selective reporting (low: seven; uncertain: two), and other bias (low: six; uncertain: three) (Figure 2). In other bias, the sample size was not calculated, and if it was judged that there was a difference in the baseline characteristics, it was assessed as uncertain [26].

### 3.3. Joint Mobilization for Individuals with Chronic Ankle Instability

In the included nine RCTs, 364 individuals with CAI participated. The interventions included JM and its sub-techniques (manipulations, non-weight-bearing and weight-bearing mobilization) in manual therapy. The duration was not determined and varied from one day to six weeks. The outcome measures included the ankle dorsiflexion range of motion (DFROM), dynamic balance (step down test, star excursion balance test, single-limb balance test), and function (American orthopedic foot and ankle society, Cumberland ankle instability tool, foot and ankle ability measure) (Table 1).

### 3.4. Effectiveness of Joint Mobilization on Ankle Dorsiflexion Range of Motion

**Figure 3:**
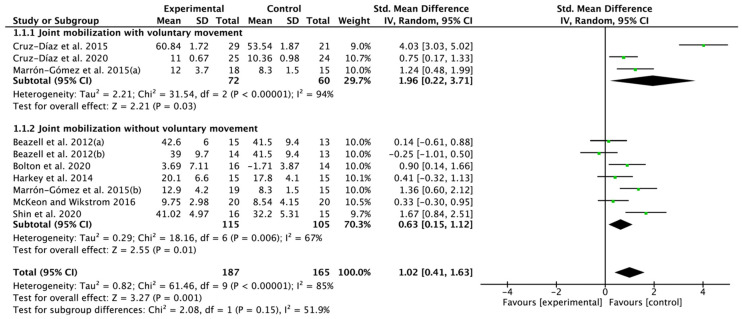
Forest plot on the effect of joint mobilization on dorsiflexion range of motion. Beazell et al. 2012(a) [18], proximal tibiofibular joint mobilization; Beazell, et al. 2012(b) [18], distal tibiofibular joint mobilization; Marrón-Gómez, et al. 2015(a) [22], weight bearing mobilization with movement; Marrón-Gómez, et al. 2015(b) [22], talocrural joint mobilization. Cruz-Díaz et al. 2015 [19], Cruz-Díaz et al. 2020 [17], Bolton et al. 2020 [23], Harkey et al. 2014 [25], McKeon and Wikstrom 2016 [21], Shin et al. 2020 [20].

Eight papers were selected from the included nine RCTs. A significant improvement was found in the results to investigate only the effect of JM, SMD = 1.02; 95% confidence interval (CI): 0.41 to 1.63; heterogeneity (χ^2^ = 61.46, df = 9, I^2^ = 85%); and overall effect (Z = 3.27, *p* = 0.001). In subgroup analysis, JM with voluntary movement showed improved results, SMD = 1.96; 95% CI: 0.22 to 3.71; heterogeneity (χ^2^ = 31.54, df = 2, I^2^ = 94%); and overall effect (Z = 2.21, *p* = 0.03), and similarly, positive benefits were also found for JM without voluntary movement, SMD = 0.63; 95% CI: 0.15 to 1.12; heterogeneity (χ^2^ = 18.16, df = 6, I^2^ = 67%); and overall effect (Z = 2.55, *p* = 0.01) (Figure 3).

### 3.5. Effectiveness of Joint Mobilization on Dynamic Balance

**Figure 4:**
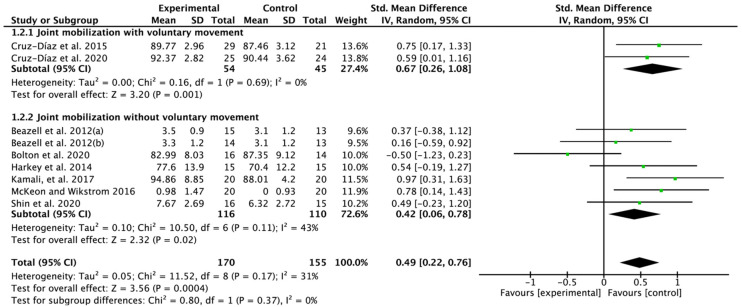
Forest plot on the effect of joint mobilization on dynamic balance. Beazell et al. 2012(a) [18], proximal tibiofibular joint mobilization; Beazell, et al. 2012(b) [18], distal tibiofibular joint mobilization. Cruz-Díaz et al. 2015 [19], Cruz-Díaz et al. 2020 [17], Bolton et al. 2020 [23], Harkey et al. 2014 [25], McKeon and Wikstrom 2016 [21], Shin et al. 2020 [20], Kamali et al. [24].

Eight papers were selected from the included nine RCTs. A significant improvement was found in the results to investigate only the effect of JM, SMD = 0.49; 95% CI: 0.22 to 0.76; heterogeneity (χ^2^ = 11.52, df = 8, I^2^ = 31%); and overall effect (Z = 3.56, *p* = 0.0004). In the subgroup analysis, JM with voluntary movement showed improved results, SMD = 0.67; 95% CI: 0.26 to 1.08; heterogeneity (χ^2^ = 0.16, df = 1, I^2^ = 0%); and overall effect (Z = 3.20, *p* = 0.001), and similarly, positive benefits were also found for JM without voluntary movement, SMD = 0.42; 95% CI: 0.06 to 0.78; heterogeneity (χ^2^ = 10.50, df = 6, I^2^ = 43%); and overall effect (Z = 2.32, *p* = 0.02) (Figure 4).

### 3.6. Effectiveness of Joint Mobilization on Function

**Figure 5:**
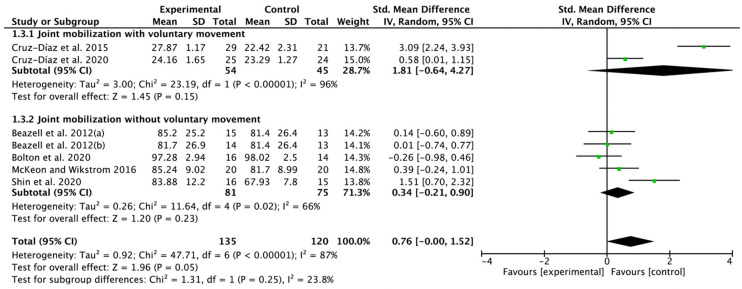
Forest plot on the effect of joint mobilization on function. Beazell et al. 2012(a) [18], proximal tibiofibular joint mobilization; Beazell, et al. 2012(b) [18], distal tibiofibular joint mobilization. Cruz-Díaz et al. 2015 [19], Cruz-Díaz et al. 2020 [17], Bolton et al. 2020 [23], McKeon and Wikstrom 2016 [21], Shin et al. 2020 [20].

Six papers were selected from the included nine RCTs. No significant improvement was found in the results to investigate only the effect of JM, SMD = 0.76; 95% CI: −0.00 to 1.52; heterogeneity (χ^2^ = 47.71, df = 6, I^2^ = 87%); and overall effect (Z = 1.96, *p* = 0.05). In the subgroup analysis, JM with voluntary movement did not show any improvement, SMD = 1.81; 95% CI: −0.64 to 4.27; heterogeneity (χ^2^ = 23.19, df = 1, I^2^ = 96%); and overall effect (Z = 1.45, *p* = 0.15), and similarly, no positive benefits were also found for JM without voluntary movement, SMD = 0.34; 95% CI: −0.21 to 0.90; heterogeneity (χ^2^ = 11.64, df = 4, I^2^ = 66%); and overall effect (Z = 1.20, *p* = 0.23) (Figure 5).

### 3.7. Publication Bias

In this review, nine studies were synthesized for systematic review and meta-analysis. Since fewer than 10 studies were synthesized according to the recommendations of the Cochrane Review, no publication bias was reported [27].

## 4. Discussion

This review synthesized and analyzed RCTs in which JM was performed as a treatment recommended as strong evidence in clinical practice guidelines for individuals with CAI. In particular, what differentiated it from the existing reviews is that we performed a subgroup analysis according to the presence or absence of voluntary movement.

The results analyzed in this review showed significant improvements in dorsiflexion ROM (DFROM) and dynamic balance, except for patient-oriented outcomes that were classified as functions. These findings suggest that patient-oriented outcomes may not be sensitive to detecting deficiencies in CAI, as reported in a systematic review of CAI in 2008 [28]. Nevertheless, some changes were observed in the results of this review (standardized mean difference = 0.76, 95% confidence interval: −0.00 to 1.52). In terms of DFROM and dynamic balance, significant improvements were also found in both JM with and without voluntary movement in the subgroup analysis. Although the differences between the studies were not confirmed, interpretation through statistical significance suggests that JM with voluntary movement may have potential advantages in dynamic balance.

Various treatment methods have been suggested for CAI. External support-type insoles plus bracing, bracing, insoles, and taping showed no significant difference compared with the control group in a systematic review [29]. Meanwhile, a review of therapeutic exercise [30] reported that it was effective for self-reported function and reinjury incidence. However, it is still concluded that no specific JM recommendations should be made [5]. Based on the results and guidelines of this review, JM should be considered an effective treatment for CAI. However, follow-ups were not performed for a long time in the included studies and they were limited to immediate effects.

A subgroup analysis was performed to quantitatively analyze the potential benefits of voluntary movements; however, no differences were found. In a previously reported RCT [31], a comparative study was performed on JM with voluntary movement (active JM) and without voluntary movement (passive JM). Both DFROM and dynamic balance showed significant improvement in the active JM group compared to the passive JM group (*p* < 0.05). Therefore, individuals with CAI differ in cortical excitability and joint laxity compared to healthy adults [32], and activation of the supplementary motor area is required [33]. In addition, as in the study of muscle activation patterns, voluntary movements are required more to increase the onset time of the tibialis anterior and peroneus longus [10]. Based on these grounds, it is considered that JM with voluntary movement should have a greater influence on decreased joint position sense than simply passive JM.

Our systematic review and meta-analysis of RCTs had the following limitations: since there are fewer than 10 synthesized studies, there is a limit to generalization; no clear protocol for JM is presented, and it is limited to carryover effects, not just immediate effects. In future experimental studies, variables related to cortical excitability will be considered appropriate to explain the mechanism of treatment. In addition, in a systematic review, it would be worthwhile to analyze the effects combined with or compared with therapeutic exercise.

## 5. Conclusions

For individuals with chronic ankle instability, joint mobilization has a limited function (patient-oriented outcome) but has positive benefits in the immediate effects on dorsiflexion and dynamic balance without any difference in voluntary movement with or without.

## Figures and Tables

**Table 1:** Characteristics of included studies.

Study	Sample Size	Duration	Intervention	Outcome	Author’s Conclusion
Beazell et al., 2012 [18]	EG1 = 15 EG2 = 14 CG = 13	Three weeks	EG1 = proximal TFJM EG2 = distal TFJM CG = no intervention	DFROM SDT FAAM	The use of a proximal or distal tibiofibular joint manipulation in isolation did not enhance outcome effects beyond those of the control group.
Bolton et al., 2021 [23]	EG = 16 CG = 14	Twice a week for six weeks	EG = TJM plus exercise CG = exercise	DFROM SEBT FAAM	The addition of MT to exercise may improve the improvement in ROM compared to exercise alone.
Cruz-Díaz et al., 2014 [19]	EG = 29 CG = 21	Twice a week for three weeks	EG = WB_MWM CG = no intervention	DFROM SEBT CAIT	Joint mobilization techniques applied to subjects suffering from CAI were able to improve ankle DFROM, postural control, and self-reported instability.
Cruz-Díaz et al., 2020 [17]	EG = 25 CG = 24	Twice a week for 12 weeks	EG = MWM plus CrossFit CG = CrossFit	DFROM SEBT CAIT	Ankle joint self-mobilization and CrossFit training were effective in improving ankle DFROM, dynamic postural control and self-reported instability in patients with CAI.
Harkey et al., 2014 [25]	EG = 15 CG = 15	One day	EG = TJM CG = no intervention	DFROM SEBT	A single joint-mobilization treatment was efficacious at restoring DFROM in participants with CAI.
Kamali et al., 2017 [24]	EG = 20 CG = 20	One day	EG = TJM CG = sham	SEBT	TJM can significantly increase the functional performance of athletes with CIA and can be an effective supplementary treatment for these subjects.
Marrón-Gómez et al., 2015 [22]	EG1 = 18 EG2 = 19 CG = 15	48 h	EG1 = WB_MWM EG2 = TJM CG = sham	DFROM	A single application of the WB-MWM or HVLA manual technique improves ankle dorsiflexion in people with CAI, and the effects persist for at least two days.
McKeon and Wikstrom 2015 [21]	EG = 20 CG = 20	Six times in two weeks	EG = TJM CG = no intervention	WBLT SLBT FAAM	Both joint mobilization and plantar massage appear to demonstrate the greatest potential to improve sensorimotor function in those with CAI.
Shin et al., 2020 [20]	EG = 16 CG = 15	Twice a week for four weeks	EG = TJM plus ATE CG = ATE	DFROM AMTI AccuSway AOFAS score	Adding HVLA to resistance exercises may be synergistically effective in improving the ankle status, pain intensity, ROM, and balance ability in ABP with CAI.

AOFAS, American orthopedic foot and ankle society; ATE, ankle therapeutic exercise; CAI, chronic ankle instability; CAIT, Cumberland ankle instability tool; CG, control group; DFROM, dorsiflexion range of motion; EG, experimental group; FAAM, foot and ankle ability measure; HVLA, high-velocity low-amplitude manipulation; MWM, mobilization with movement; SDT, step down test; SEBT, star excursion balance test; SLBT, single-limb balance test; TFJM, tibiofibular joint manipulation; TJM, talocrural joint mobilization; WB, weight bearing; WBLT, weight bearing lunge test.

## Data Availability

Not applicable.
